# Heterogeneity of Blood Vessels and Assessment of Microvessel Density-MVD in Gingivitis

**DOI:** 10.3390/jcm11102758

**Published:** 2022-05-13

**Authors:** Ciprian Roi, Pușa Nela Gaje, Raluca Amalia Ceaușu, Alexandra Roi, Laura Cristina Rusu, Eugen Radu Boia, Simina Boia, Ruxandra Elena Luca, Mircea Riviș

**Affiliations:** 1 Department of Anesthesiology and Oral Surgery, Multidisciplinary Center for Research, Evaluation, Diagnosis and Therapies in Oral Medicine, “Victor Babeș” University of Medicine and Pharmacy, 300041 Timisoara, Romania; ciprian.roi@umft.ro (C.R.); rivis.mircea@umft.ro (M.R.); 2 Department of Microscopic Morphology and Histology, Angiogenesis Research Center, “Victor Babeș” University of Medicine and Pharmacy, 300041 Timisoara, Romania; gaje.nela@umft.ro (P.N.G.); ra.ceausu@umft.ro (R.A.C.); 3 Department of Oral Pathology, Multidisciplinary Center for Research, Evaluation, Diagnosis and Therapies in Oral Medicine, “Victor Babeș” University of Medicine and Pharmacy, 300041 Timisoara, Romania; laura.rusu@umft.ro; 4 Department of Ear Nose and Throat, “Victor Babeș” University of Medicine and Pharmacy Timisoara, 300041 Timisoara, Romania; eugen.boia@umft.ro; 5 Department of Periodontology, “Victor Babeș” University of Medicine and Pharmacy Timisoara, 300041 Timisoara, Romania; simina.boia@umft.ro; 6 Department of Oral Rehabilitation and Dental Emergencies, “Victor Babeș” University of Medicine and Pharmacy, 300041 Timisoara, Romania; luca.ruxandra@umft.ro

**Keywords:** gingivitis, MVD, angiogenesis, CD34

## Abstract

Gingivitis is a very common oral disease highly prevalent in adults that, if left untreated, can progress to periodontitis. It involves a complex and slow interaction between the host response and the oral microbiome represented by the dental plaque. The inflammation of the gingiva is associated with the activation of pathological angiogenesis and the existence of a high number of newly formed blood vessels quantified as microvessel density (MVD). The present study includes a number of 51 gingival biopsies from patients with different gingival indexes (GI): GI = 0, *n* = 12; GI = 1, *n* = 15; GI = 2, *n* = 16; and GI = 3, *n* = 8, processed and stained with the routine hematoxylin–eosin method. The inflammatory infiltrate was scored, the blood vessels were detected with anti-CD34 antibody, and MVD was determined. Inflammatory changes were observed in 39 of the 51 cases included in our study. CD34 + vessels with normal morphological appearance were observed in all 12 cases of health gingiva. In cases of inflammatory lesions, the morphology of the blood vessels showed changes with the evolution of gingival lesions. In severe inflammation, a particular aspect was observed in the vessels, such as the presence of the phenomenon of intussusception. MVD increases with the severity of gingival lesions, with the highest density being observed in severe inflammation.

## 1. Introduction

Periodontal health is defined by Chapple as a “state free from inflammatory periodontal disease that allows an individual to function normally and avoid consequence (mental or physical) due to current or past disease” [1]. The periodontal health of an individual can be assessed by the clinical absence of diseases such as gingivitis, periodontitis, or other periodontal disorders.

On the other hand, periodontal disorders are very common and may impact up to 90% of the world’s population. Gingivitis is a pathological condition often associated with bacterial biofilm that is generally reversible upon a rigorous reinstatement of oral hygiene procedures [2]. Despite the fact that it is considered to be the “route” towards the development of periodontitis, affecting a considerably high number of patients, this pathology is often disregarded [3,4,5].

The study conducted by Lang et al. [3] is based on identifying the presence of gingivitis as a key risk factor in the incidence of periodontal disease, revealing their association in approximately 37% of the gingivitis cases and showing a definite progression to periodontitis and loss of teeth thereafter. A patient diagnosed with gingivitis can revert to a complete state of oral health, while once diagnosed with periodontitis, even following successful therapy, further lifelong supportive care is required to prevent the recurrence of the disease [6]. The heterogeneity of this condition is also highlighted during the treatment protocol, revealing a percentage of the diagnosed gingivitis cases that do not respond properly to standard mechanical treatment [7] and the healing of the tissue exhibiting normal biological conditions is absent [8].

The classification of gingival diseases is made by taking into consideration the presence or absence of dental plaque. Dental plaque-induced gingival diseases may occur on a periodontium with no attachment loss or with attachment loss that is stable and not progressing [9].

Angiogenesis in the oral mucosa diseases, and implicitly gingivitis, is a negative prognostic factor that potentiates the manifestations of the disease and worsens its progression. The process is defined as the formation of new blood vessels from pre-existing ones. It involves endothelial cell migration and proliferation, and the further formation and organization of tubular structures that in time will bond, resulting in a final form of stable blood vessels. Through this process, the neoformation vessels bring pro-inflammatory cells and mediators, but also oxygen and nutrients to the inflamed tissues [10]. The inflammation of the gingiva, which is the main mechanism for periodontal lesions, is associated with the activation of pathological angiogenesis and the existence of a high number of newly formed blood vessels quantified as a microvessel density (MVD) [11].

The transition from gingivitis without bone loss to periodontitis is explained by the propagation of the inflammatory response, correlated with the failure of the innate inflammation resolving mechanisms. In the end, it results in the chronicity of the inflammatory lesion, which is histologically characterized by the present repair mechanisms signs (angiogenesis and fibrosis) occurring concurrently with inflammation [12]. It plays a significant role as well in bone regeneration, contributing to the inflammatory and regenerative phase of the alveolar bone. The microvessel density evaluation targets the acknowledgment of the angiogenesis level and its implication in multiple inflammatory conditions has been widely accepted. 

Different markers (monoclonal antibodies) such as CD34, CD31, CD105, vascular endothelial growth factor (VEGF), and beta fibroblast growth factor (FGFβ) are used to measure microvessel density (MVD) in each microscopic field [13,14]. 

CD34 is a monomeric transmembrane glycoprotein with a molecular weight of 110–120 KD, which has a high sensitivity and moderate specificity. The anti-CD34 antibody, the QBEnd10 clone, is the most widely used immunohistochemical marker in the study of tumor angiogenesis and microvessel density, being positive on paraffin sections, ice, and Western blotting techniques. CD34 expression is frequently used to evaluate MVD, unlike CD31 which, in addition to being present in endothelial cells, is also localized in the macrophages [15,16].

In the scientific literature, existing studies regarding the angiogenesis evaluation in gingivitis are very limited, with the emphasis being on periodontitis and oral neoplasms. Starting from this aspect, in the present study, we aim to evaluate by immunohistochemical methods the morphology of blood vessels and microvessel density in inflammatory gingival lesions compared to normal, healthy gingiva. The quantification of MVD was established by the number of vessels in the inflammatory infiltrate, in the surrounding stroma, and also in the epithelium. Taking into consideration that gingivitis and its persistence influences the further development of periodontitis, the assessment of the microvessel density and heterogeneity in this stage could be an evolution marker in the possible progression of this disease. Our study brings a new point of view regarding the histologic aspects of gingivitis without bone loss and can promote further studies to establish the mechanism of clinical aggravation of gingivitis and transformation in periodontitis.

## 2. Materials and Methods

Our study was approved by the Ethics Committee of “Victor Babeș” University of Medicine and Pharmacy Timișoara (no.12/2021) and patients agreed and signed an informed consent form that followed the guidelines of the Declaration of Helsinki.

### 2.1. Patients’ Data

In this study, we included patients based on the following inclusion and exclusion criteria.

Inclusion criteria:

- Age: 18–60 years;
- Both males and females;
- Diagnosed gingivitis with no attachment loss;
- Periodontal and gingivitis-free patients (for the control group);
- No known general comorbidities;
- Non-smokers;
- Non-alcohol drinkers.

Exclusion criteria:

- Gingivitis modified by systemic factors, such as medication;
- Periodontitis;
- Patients with periodontal therapies;
- Patients with medication.

After the application of the inclusion and exclusion criteria, a total of 51 patients were included in the study, 28 females—54.9% and 23 males—45.09%. The Gingival Index (GI) used for the assessment of the gingivitis had the following values: GI = 0, *n* = 12; GI = 1, *n* = 15; GI = 2, *n* = 16; and GI = 3, *n* = 8, with a mean value of 1.82. The biopsy samples taken from healthy patients that were included in the control group were based on the following clinical criteria: the absence of gingival erythema, no bleeding while probing, no clinical visible plaque deposits, and a probing depth within 2 mm.

**Figure 1:**
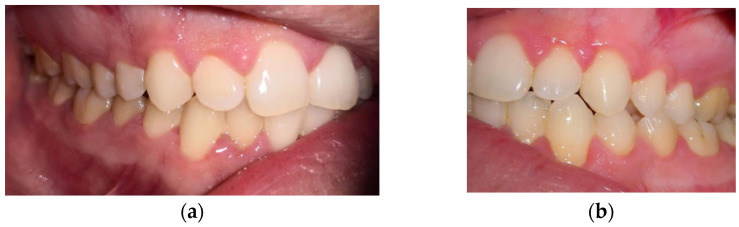

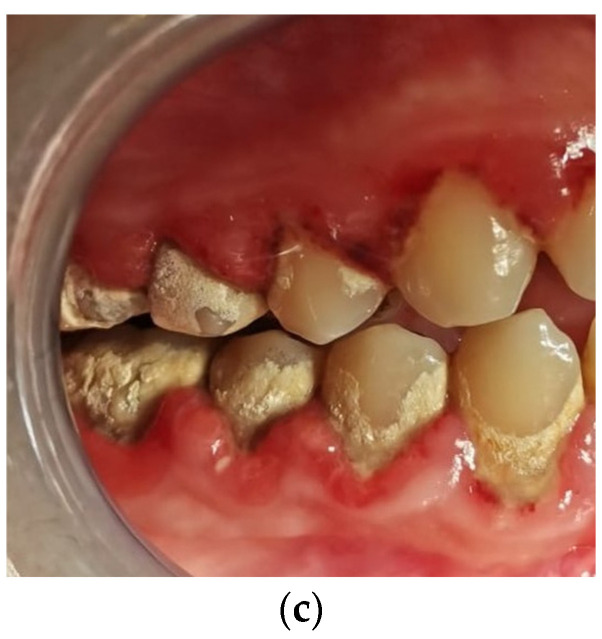
Clinical images of gingivitis: (**a**) mild inflammation-GI = 1; (**b**) moderate inflammation-GI = 2; and (**c**) severe inflammation-GI = 3.

In Figure 1, the clinical assessment of gingivitis is presented in the three stages of inflammation.

The present study included a total of 51 gingival biopsies that were processed according to the standard histological technique. The gingival biopsies were obtained from the interdental papilla between the mandibular first and second premolar. A biopsy was taken from each patient and washed with buffer saline.

### 2.2. Primary Processing

Gingival biopsies were fixed in 10% buffered formalin for 48 h and then embedded in paraffin using the standard histological technique. The primary processing was completely standardized using the Shandon embedding center (Thermo-Shandon, Runcorn, Chershire, UK). Five micrometer-thick sections were prepared for each case and were stained with the routine hematoxylin–eosin method. These slides were used to analyze the morphological changes of the epithelium and to evaluate the density of the inflammatory infiltrate. Additional slides were prepared and selected for the immunohistochemical study.

#### Scoring

The inflammatory infiltrate was scored as 0 (absent), value 1 (isolated inflammatory cells, less than 10 inflammatory cells/microscopic field, and low inflammation), value 2 (aggregates of inflammatory cells in the lamina propria only and moderate inflammation), and value 3 (aggregates of inflammatory cells in the lamina propria associated with intraepithelial lymphocytes and severe inflammation).

### 2.3. Imunohistochimical Technique 

Blood vessels were detected with anti-CD34 antibody—monoclonal mouse, clone QBEnd 10, ready to use, and Leica Bond (Leica Biosystem, Newcastle Ltd., Newcastle upon Tyne, UK). A heat-induced epitope retrieval with Bond Epitope Retrieval Solution 1 citrate buffer (pH 6.0) (Leica Biosystems, Newcastle Ltd., Newcastle upon Tyne, UK) for 30 min was applied. Endogenous peroxidase was blocked for five minutes with 3% hydrogen peroxide and followed by incubation with the primary antibody for 30 min. The Bond Polymer Refine Detection System (Leica Biosystems, Newcastle upon Tyne, UK) was used to develop the immunohistochemical reaction and the final product was visualized with 3,3′ diaminobenzidine dihydrochloride (DAB). The chromogen was applied for 10 min and the hematoxylin was used for 5 min for the counterstaining. The full immunohistochemical procedure was performed with a Leica Bond-Max (Leica Biosystems, Newcastle upon Tyne, UK) autostainer.

### 2.4. Staining Interpretation

All the sections were examined using a Nikon Eclipse 600 optical microscope. Initially, the examination was made using the ×100 lens in order to identify the most intensely positive areas for CD34. Vessel quantification was performed using the ×40 lens. The area of each field was approximately 0.2 mm^2^. The endothelial cells colored with brown CD34 (CD34-positive) that formed a cluster of endothelial cells with a lumen were considered to be blood vessels. Single CD34-positive endothelial cells were also included in the count. The blood vessels with a muscle wall were excluded. The three fields with the largest number of blood vessels were chosen and then examined from left to right, avoiding counting the vessels from the same areas. The arithmetic mean of the total number of vessels encountered in the three examined fields was performed, representing the final result. The statistical evaluation was made by SPSS 17 software (IBM Analytics, Armonk, NY, USA) and a *p*-value of <0.05 was considered statistically significant.

## 3. Results

**Figure 2:**
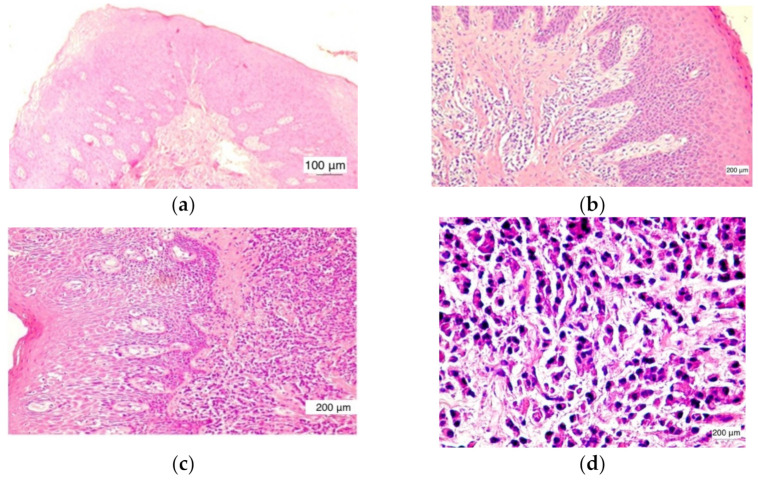
Different hematoxylin–eosin microscopic images for assessment of the morphological changes of the epithelium evaluation of the density of the inflammatory infiltrate: (**a**) gingiva without inflammatory changes GI = 0, ×200 magnification; (**b**) inflammatory infiltrate in the lamina propria with focal distribution, epithelium without modification GI = 1, ×200 magnification; (**c**) inflammatory infiltrate in the lamina propria, intraepithelial lymphocytes GI = 2, ×200 magnification; and (**d**) inflammatory infiltrate with polymorphic cellularity GI = 3, ×400 magnification.

Microscopically, in normal healthy gingiva (*n* = 12) there were no inflammatory changes present. The normal histological structure of the gingival mucosa was preserved. It was noticed that a parakeratinized stratified squamous epithelium and lamina propria were composed of dense irregular connective tissue (Figure 2a).

In the cases evaluated with a score value of +1, low inflammation, the inflammatory infiltrate was found in the lamina propria with focal distribution, but also as isolated inflammatory cells. The covering epithelium had no changes and intraepithelial lymphocytes were very rare (Figure 2b).

In the cases evaluated with a score value of +2, moderate inflammation, the inflammatory infiltrate was noticed in the lamina propria over extended areas, reaching as far as the covering epithelium. Basal cell layer hyperplasia was found in the covering epithelium (Figure 2c).

The cases evaluated with a score value of +3 were characterized by severe inflammation. The aggregates of inflammatory cells in the lamina propria distributed over large areas associated with numerous intraepithelial lymphocytes were noticed. A tendency of inflammatory infiltrate distribution around the blood vessels was found. The presence of large amounts of lymphocytes in the covering epithelium induced an alteration of its structure. Thus, a hyperplasia of basal and parabasal cells was observed (Figure 2d).

Inflammatory changes were observed in 39 of the 51 cases included in our study. The inflammatory infiltrate consisted mainly of lymphocytes, macrophages, and neutrophilic granulocytes. Rarely contained eosinophilic granulocytes and plasma cells were also identified (Figure 2c).

**Figure 3:**
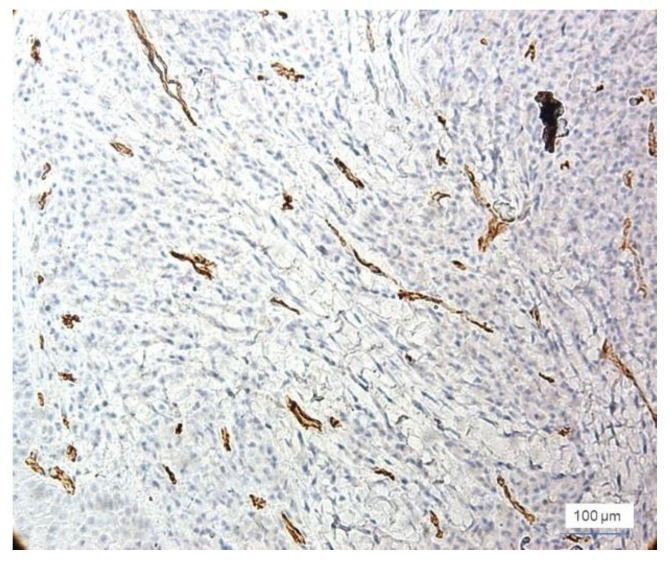
Severe inflammation marked vascular polymorphism, small vessels with or without lumen narrow, anti-CD34 immunohistochemical staining, and DAB chromogen, ×100 magnification.

Out of the 39 cases with inflammatory lesions, 15 cases showed mild inflammation (G I = 1, score value 1), 16 cases showed moderate inflammation (GI = 2, score value 2), and 8 cases had severe inflammatory lesions (GI = 3, score value 3), as found by clinical examination. The microscopic features of the normal gingiva, mild, moderate, and severe periodontal disease are shown in Figure 2. CD34 + vessels with a normal morphological appearance were observed in all 12 cases of healthy gingiva. The identified vessels are predominantly capillary and distributed throughout the gingival lamina, including the papillae to the proximity of the surface epithelium. In all of these cases, the blood vessels were small in diameter and all had a lumen. In cases of inflammatory lesions, the morphology of the blood vessels showed changes in accordance with the evolution of the periodontal lesions. Thus, both small-caliber vessels with a very narrow lumen delimited by proliferative endothelial cells and small vessels without a lumen were observed (Figure 3).

Small narrow-lumen vessels have been observed in large amounts in gingival lesions with moderate and severe inflammation. In the cases of severe inflammation, vascular structures in the form of cords with a tendency to form a lumen were encountered.

**Figure 4:**
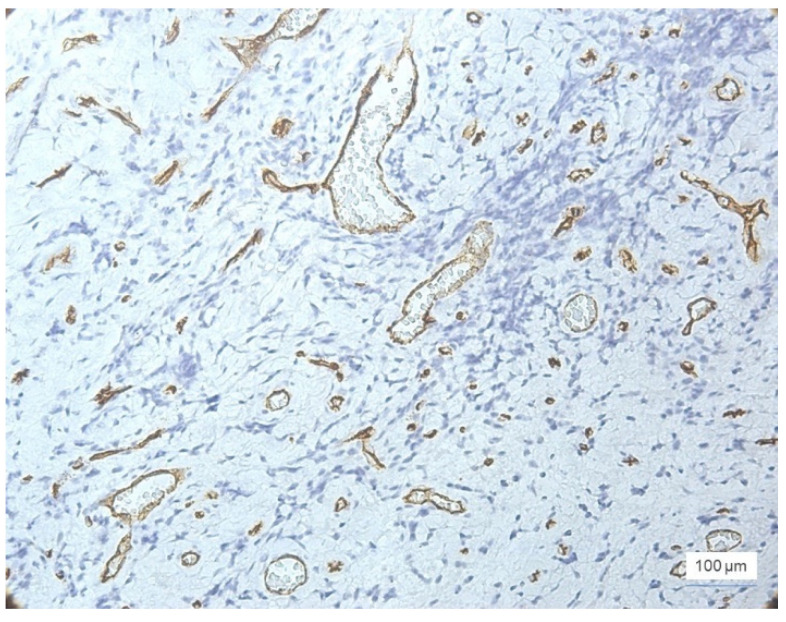
Large dilated and branched vessels, anti-CD34 immunohistochemical staining, and DAB chromogen, ×200 magnification.

Most cases with severe inflammation showed extensive areas of bleeding. In these cases, we identified large dilated vessels with dilated stasis and numerous branches, suggesting the activation of endothelial cells (Figure 4).

**Figure 5:**
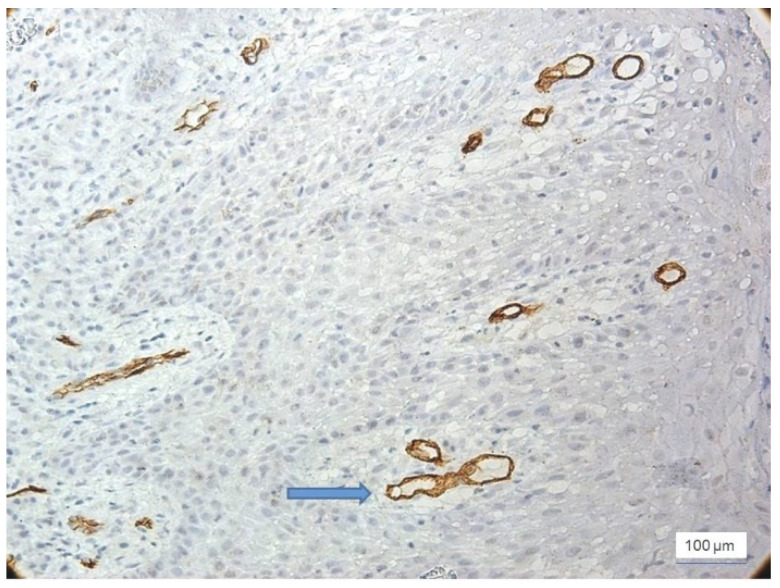
Severe inflammation, intussusception (arrow), intraluminal bridges dividing the blood vessel, anti-CD34 immunohistochemical staining, and DAB chromogen, ×200 magnification.

In severe inflammation, a particular aspect was observed in the vessels: the presence of the phenomenon of intussusception, characterized by invagination of the vascular wall with the formation of intraluminal bridges that divided the initial vessel into two smaller vessels (Figure 5).

All these aspects related to the changes found in the morphology of the blood vessels in cases with inflammatory changes suggest that the initiation of angiogenesis in moderate and severe periodontal lesions was present.

Microvessel density (MVD) was quantified and the number of CD34 + vessels near the inflammatory infiltrate, in the surrounding stroma, and in the epithelium was observed. In all cases of inflammatory lesions, an increase in MVD was observed in proportion to the severity of the inflammation. Regardless of the severity of inflammation, the MVD was significantly increased in the stroma compared to the MVD in the infiltrate. Thus, in mild inflammation, the total number of vessels was between 12 and 35 vessels/microscopic field. Only two cases with mild inflammation showed a higher MVD (approximately 46 vessels/field).

**Figure 6:**
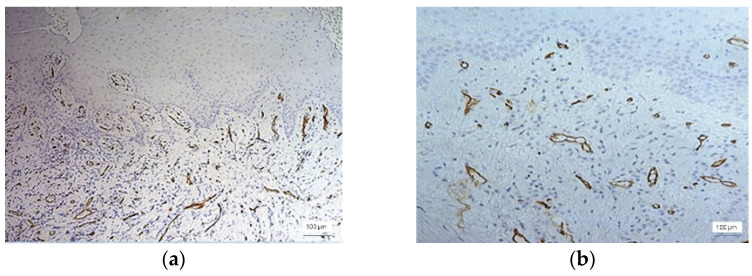
Moderate inflammation, GI = 2, and increased microvessel density at the stromal level, ×100: (**a**) stromal vascular polymorphism and intussusception, ×200, and (**b**) anti-CD34 immunohistochemical staining and DAB chromogen.

In moderate inflammation, MVD was slightly increased compared to the cases with mild inflammation. The number of vessels was 15–49/field, with the majority being identified in the stroma around the inflammatory infiltrate (Figure 6).

**Figure 7:**
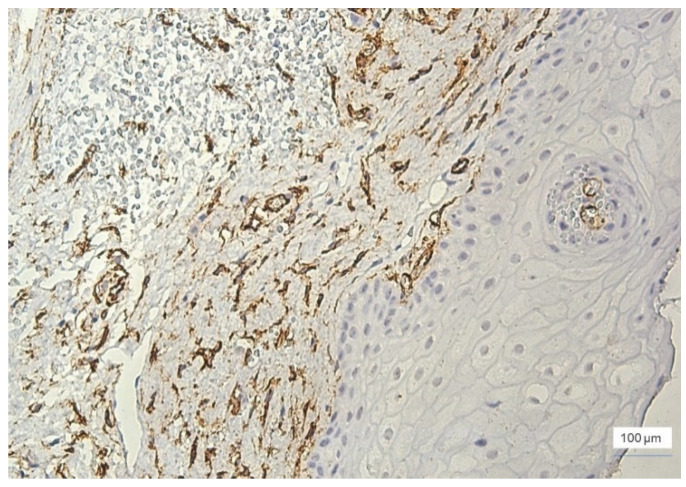
Severe inflammation, GI = 3, increased microvessel density at the stromal level, vascular polymorphism, anti-CD34 immunohistochemical staining, and DAB chromogen, ×100.

As expected, the highest values for MVD were observed in cases of severe inflammation, where the total number of vessels was between 35–89/field, most of them being observed in the stroma (Figure 7). Two cases with severe inflammation showed a lower MVD (20–25 vessels/field).

**Figure 8:**
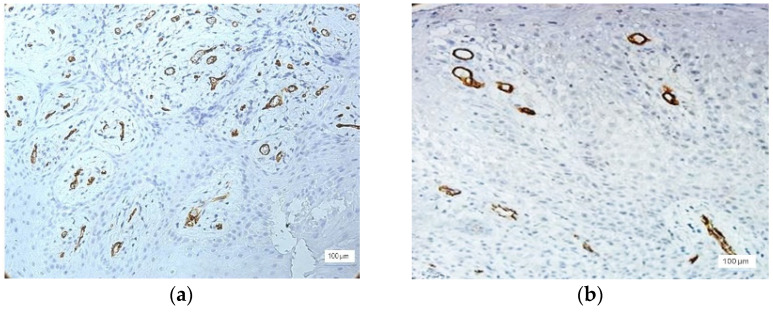

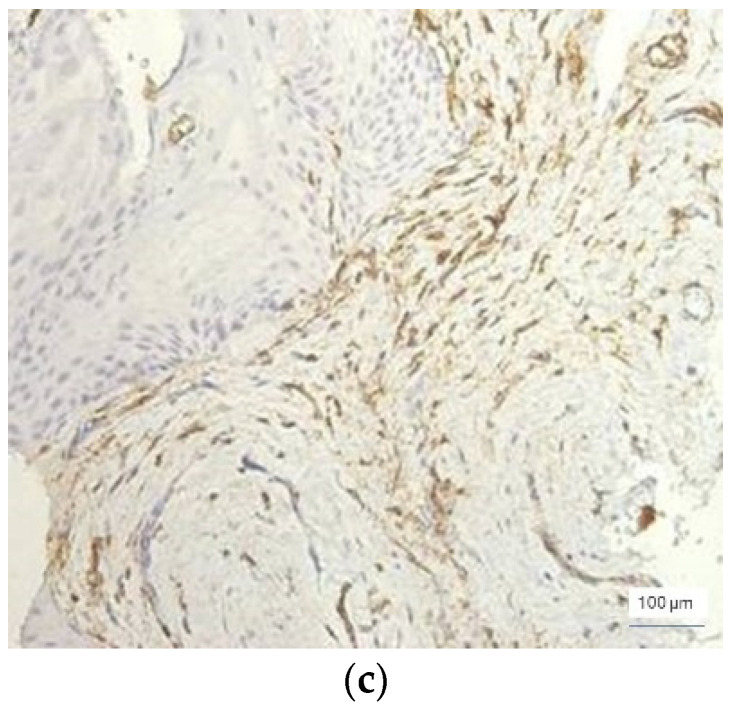
(**a**–**c**) Capillaries in the basal layer of the epithelium, anti-CD34 immunohistochemical staining, and DAB chromogen, ×200 magnification.

The presence of small capillary vessels was observed in the gingival epithelium, as well as in the cases with severe inflammation (Figure 8). Capillaries have been identified in the basal layer of the gingival epithelium.

## 4. Discussion

According to the new classification scheme for periodontal and peri-implant diseases and conditions established at the World Workshop on the Classification of Periodontal and Peri-implant Diseases in 2017, gingivitis is defined as a site-specific inflammatory condition initiated by dental biofilm accumulation and characterized by edema and erythema of the gingival tissue with the absence of periodontal attachment loss [6,17]. Generally, it is a painless condition, which rarely leads to spontaneous bleeding. The tissue changes are completely reversible, once the dental biofilm is removed. Gingivitis is a precursor of periodontitis, which is characterized by gingival inflammation combined with connective tissue attachment and bone loss [17].

The development of new vessels from pre-existing vessels, called angiogenesis, is a complex process involving the remodeling of the extracellular matrix, the migration and proliferation of endothelial cells, and the morphogenesis of microvessels. Angiogenesis is often a significant and independent prognostic indicator for both overall survival rates and diseases [18]. Angiogenesis is one of the most well-known stromal factors involved in tumor progression as well as in inflammatory lesions, such as periodontal lesions. It has been extensively investigated in various tumors, such as breast carcinoma, hepatocellular carcinoma, astrocytoma, cervical carcinoma, and ovarian carcinoma, but also in several lesions and odontogenic tumors such as ameloblastoma [19]. 

As a result of the interaction between the gingival epithelium and the stromal compartment, angiogenesis is an early and constant process in gingivitis and periodontal diseases, occurring at all stages [20]. Angiogenesis and the dilation of capillaries appear to be characteristic of the vascular response in chronic inflammation, and apart from gingivitis and periodontitis, are also reported in rheumatoid arthritis, psoriasis, and other chronic inflammatory lesions [21].

Persistence of the gingival inflammation under the presence of various local and general risk factors progresses to the destruction of the underlying connective tissue and eventually to the destruction of the alveolar bone [22]. 

The proximity of this thick inflammatory infiltrate to the sulcular epithelium, which is in direct contact with the bacterial plaque irritant, demonstrates the importance of the bacterial plaque which plays a key role in the initiation and progression of gingivitis. A single kind of organism is not involved in the change from a healthy periodontium to an inflammatory periodontium. In periodontal diseases, the microbial communities are dysbiotic, with uncontrolled microbial species composition and abundance resulting in a pathogenic situation. Despite the fact that the Gram-negative organisms, “red complex” are associated with periodontal disease, they are also found at low levels in healthy patients without periodontal disease, suggesting that they are pathobionts rather than pathogens [21].

Periodontitis shares many of the same histological characteristics as gingivitis, with the exception that the connective tissues at the base of the gingival sulcus are destroyed, resulting in the formation of a deep periodontal pocket bordered by epithelium. In our findings, the inflammatory infiltrate consisted mainly of lymphocytes, macrophages, and neutrophilic granulocytes-PMN, and more rarely consisted of eosinophilic granulocytes and plasma cells. Vascular alterations appear to either assist or inhibit PMN activity, influencing the progress of the disease. In periodontitis, high endothelial cells are engaged with PMN rather than lymphocyte emigration, contrary to most circumstances [23]. 

The findings obtained in our study sustain the heterogeneity of the angiogenic process dependent on the level of gingiva inflammation. In tissues, the degree of angiogenesis can be evaluated by microvessel density (MVD) using an antibody against CD34, a glycosylated transmembrane protein present on progenitor endothelial cells [24]. Antibodies to the CD34 molecule are widely used for immunohistochemical staining of gingival microvessels MVD found in the early stages of gingivitis and may be considered the first step of a complex process with damages countable depending on the gingival index. MVD measurement is a widely known predictor of tumor growth, metastasis, and patient survival rate and correlates with tumor aggression [19]. In addition to this, MVD is also associated with the progression of gingivitis lesions as observed in our study. Increased MVD could cause a higher apport of different cytokines, adhesion molecules, and other inflammation factors. In gingivitis and periodontitis, the transport of inflammatory cells, nutrients, and oxygen caused by angiogenesis could enhance the severity of the inflammation [24].

Vascular changes are essential to the initiation of both acute and chronic inflammation, and blood flow is essential to its resolution. Inflammation begins with vasodilation, increasing circulation, and increased vascularization into the area. The progressive disorder of affected gingiva perfusion and oxygenation, the presence of increased vascular permeability, and functional failure of the microvascular system are the main processes involved in disease progression [25,26].

In the development of gingivitis, an important role is played by the vascular endothelial growth factor (VEGF), a 45-kd homodimeric proinflammatory glycoprotein that causes vascular permeability and angiogenesis. This protein seems to be involved in the onset and progression of gingivitis and periodontitis, mainly promoting the vascular network expansion generally observed in inflammation [27]. Endothelial cells produce proteases and plasminogen activators in response to VEGF, which break down the vascular basement membrane and allow endothelial cells to proliferate and migrate [28]. The double correlation of intraepithelial-increased MVD with VEGF may be considered a unique and specific feature of mild gingivitis lesions progressing to moderate lesions, indicating the initiation of the ‘vascularization’ phenomenon, which refers to the acquisition of new blood vessels by the affected gingival epithelium [11]. Lucarini et al. [29] in their study concluded the fact that the gingival mucosa affected by an inflammatory condition has significantly higher levels of VEGF and MVD compared to the healthy mucosa. VEGF has a direct implication in the vascular network, it increases the tissue edema, and influences the blood flow by decreasing it, suggesting a high implication in the etiology of gingivitis.

Acknowledging the heterogeneity of the blood vessels and the microvessel density in gingivitis is an important aspect of fully understanding the evolution of this disease and its possible progress towards periodontitis with high clinical relevance. The vascular changes encountered in gingivitis could represent a viable correlation in anticipating the incidence of periodontal involvement. 

## 5. Conclusions

The modifications observed in patients presenting gingivitis indicate the presence of angiogenesis, an aspect suggested by increased vascular polymorphism, the phenomenon of intussusception, and finally, the acquisition of vessels in the gingival epithelium. MVD increases with the severity of gingival lesions, with the highest density being observed in severe inflammation.

## Data Availability

Data sharing is not applicable to this article.
